# Snapshot study of canine distemper virus in Bangladesh with on-site PCR detection and nanopore sequencing

**DOI:** 10.1038/s41598-024-59343-6

**Published:** 2024-04-22

**Authors:** Zsófia Lanszki, Md. Shafeul Islam, Md. Foisal Shikder, Md. Jalal Uddin Sarder, Shahneaz Ali Khan, Sharmin Chowdhury, Md. Nurul Islam, Zsófia Tauber, Gábor Endre Tóth, Ferenc Jakab, Gábor Kemenesi, Sazeda Akter

**Affiliations:** 1 National Laboratory of Virology, Szentágothai Research Centre, University of Pécs, Pecs, Hungary; 2 Institute of Biology, Faculty of Sciences, University of Pécs, Pecs, Hungary; 3 Faculty of Veterinary and Animal Sciences, University of Rajshahi, Rajshahi, Bangladesh; 4 Department of Medicine and Surgery, Faculty of Veterinary Medicine, Chattogram Veterinary and Animal Sciences University, Chattogram, Bangladesh; 5 Department of Physiology Biochemistry and Pharmacology, Chattogram Veterinary and Animal Sciences University, Chattogram, Bangladesh; 6 Department of Pathology and Parasitology, Faculty of Veterinary Medicine, One Health Institute, Rajshahi, Bangladesh; 7 Department of Forest and Wildlife Ecology, Wisconsin Cooperative Wildlife Research Unit, University of Wisconsin–Madison, Madison, USA; 8 School of Biomedical Sciences, University of Plymouth, Plymouth, PL4 8AA UK

**Keywords:** Virology, Viral infection, Diseases

## Abstract

Canine distemper virus (CDV) is a highly contagious virus that affects domestic and wild animals, causing severe illness with high mortality rates. Rapid monitoring and sequencing can provide valuable information about circulating CDV strains, which may foster effective vaccination strategies and the successful integration of these into conservation programs. During two site visits in Bangladesh in 2023, we tested a mobile, deployable genomic surveillance setup to explore the genetic diversity and phylogenetic patterns of locally circulating CDV strains. We collected and analysed 355 oral swab samples from stray dogs in Rajshahi and Chattogram cities, Bangladesh. CDV-specific real-time RT-PCR was performed to screen the samples. Out of the 355 samples, 7.4% (10/135) from Rajshahi city and 0.9% (2/220) from Chattogram city tested positive for CDV. We applied a real-time RT-PCR assay and a pan-genotype CDV-specific amplicon-based Nanopore sequencing technology to obtain the near-completes. Five near-complete genome sequences were generated, with phylogenetic relation to the India-1/Asia-5 lineage previously identified in India. This is the first study to provide genomic data on CDV in Bangladesh and the first demonstration of a mobile laboratory setup as a powerful tool in rapid genomic surveillance and risk assessment for CDV in low resource regions.

## Introduction

Canine distemper virus (CDV) [virus species name: *Morbillivirus canis*] is a negative sense, ssRNA virus, taxonomically included within the genus *Morbillivirus* of the family *Paramyxoviridae*. There are multiple members in this genus with significant impact on animal or human health, such as the peste des petits ruminants virus (PPRV), measles virus (MV), rinderpest virus (RPV) or feline morbillivirus (FeMV)^1^. Canine distemper virus is a highly contagious virus that causes severe illness and mortality rates in a wide range of domestic and wild animals worldwide^2,3^. The virus spreads primarily through direct contact between animals, transmitted by body fluids such as respiratory droplets, conjunctival discharges, saliva, urine, and feces^4^. Severe cases of CDV can lead to death, especially if the infection affects the central nervous system. In contrast, if dogs develop a strong immune response they can recover completely from the infection^2,4^. CDV infections depend of the virulence of the virus strain, host age and immune status, secondary infections, frequency of reinfections with CDV, vaccination status and a number of environmental and behavioural conditions^5,6^.

The H gene of CDV shows a genetic variation that follows mostly geographic pattern, allowing for the classification of multiple distinct genetic lineages. Besides the hemagglutinin (H) gene classification, analysing the complete CDV genome provides a better understanding of viral genetics, possible interspecies transmission and especially for genomic recombination^7^. In Asia, several lineages of CDV have been classified so far, namely as Asia-1, Asia-2, Asia-3, Asia-4 and Asia-5 lineages^8–12^. Moreover, more recently novel lineages were identified based on amino acid sequence divergence level from known CDV lineages. A recent example is the Asia-6 lineage, discovered in red panda (*Ailurus fulgens*) in 2018 from China^13^. In the South Asia region, particularly in India, CDV were reported in multiple studies^14–18^. In addition to already known lineages and similarly to the trend in Asia where multiple, genetically divergent lineages are known, the presence of a novel genetic lineage of CDV, the India-1/Asia-5 lineage was reported in India. Based on this report, this particular CDV lineage is circulating among the stray dog population^19^.

The high population density of humans, along with the high number of stray dogs, increases the risk of CDV spillover to endangered wildlife species. There are multiple reports in the region, such as the epizootic event among Asiatic Lions (*Panthera leo persica*) and leopards (*Panthera pardus*) in 2018 in India. During this study, the India-1/Asia-5 lineage was identified by sequencing, supporting the idea of spillover events through the stray dog and wildlife interface^20^. In Bangladesh, the presence of CDV is known by multiple studies, but these studies are limited to rapid antigenic tests, clinical diagnosis or PCR, and no sequence data is available to date^21–27^.

In many developing countries, especially in South Asia where human overpopulation is significant, the plight of stray dogs is a pressing issue, as they face harsh living conditions, malnutrition, and limited access to vaccination programmes. Adopting stray dogs, implementing sterilization programmes, and providing access to vaccinations are vital steps to curbing overpopulation and mitigating disease transmission^28^. Considering that, CDV has a higher rates of cross-species transmission potential and can cause outbreaks^29^, by reducing the incidence of CDV infections in domestic animals, vaccination and other prevention programmes indirectly contributes to controlling the transmission of the virus in both captive and wild animal populations^12^. From a conservation perspective, there are several wild and captive endangered animal species such as Bengal tiger (*Panthera tigris tigris*) and Indian leopard (*Panthera pardus fusca*) in Bangladesh that are at clear risk to CDV infection^30^. Maintaining proper surveillance programs coupled with vaccination campaigns in contact dogs may greatly reduce the conservational risk toward these animals^31^.

Surveillance activities in low-resource areas are often challenging, but modern and deployable diagnostic or sequencing techniques can overcome these limitations. The utilization of diagnostic and sequencing capabilities for responding to human outbreaks has been increasingly documented with encouraging results^32–34^. However, their application for animal health concerns remains neglected.

Our main goal was to demonstrate the mobile laboratory setup as a powerful tool in rapid risk assessment, genomic surveillance, and rapid diagnostics for canine distemper virus in stray dogs of Bangladesh. We performed our work outside the capital, in two distant country cities, Rajshahi and Chattogram where animal health diagnostic possibilities are more limited compared to the capital. A major component of the work was the generation of viral genomic data by sequencing on site and provide the first sequence data of CDV from Bangladesh. Our objective is to emphasize mobile laboratory solutions as a viable tool to tackle research and diagnostic challenges in animal health.

## Results

### PCR screening and sequencing

Overall, 3.4% (12/355) of sampled animals tested positive for CDV by Real-Time RT-PCR assay (Table 1). During the first site visit, in Rajshahi city, in February we detected CDV RNA in the 7.4% (10/135) of the swab samples, whilst in Chattogram city in March (0/122) all swabs were negative. During the second site visit in June, we detected CDV RNA with 2.0% (2/98) positivity rate in the samples from Chattogram city. All tested animals were asymptomatic at the time of sampling.

**Table 1:** Summary data of CDV positive samples.

Collection date	City	Accession number	RT-PCR Ct value	mPCR cycle number^a^
26.02.2023	Rajshahi	–	37.51	35
26.02.2023	Rajshahi	–	41.65	35
26.02.2023	Rajshahi	–	35.94	35
26.02.2023	Rajshahi	–	44.01	35
28.02.2023	Rajshahi	–	36.24	35
28.02.2023	Rajshahi	OR880598	32.33	35
28.02.2023	Rajshahi	OR880599	27.52	30
28.02.2023	Rajshahi	OR880600	28.27	30
28.02.2023	Rajshahi	–	37.98	35
28.02.2023	Rajshahi	OR880601	35.45	35
20.06.2023	Chattogram	–	34.91	35
20.06.2023	Chattogram	OR880602	31.94	35

^a^Number of multiplex PCR cycles during the amplicon-based NGS sequencing protocol.

We managed to sequence 5 near-complete genome sequences from the 12 positive samples with Nanopore sequencing on-site. Similarly, to previous studies we observed a correlation of low-coverage or partial genome sequences with higher qRT-PCR Ct values^7,35,36^. These low-coverage and partial sequences are most probably due to the low virus titer in the original sample, and due to low quality, these cannot be interpreted in further genomic analyses.

Obtained sequences were deposited in GenBank (accession numbers OR880598–OR880602). Based on the GenBank BLASTn similarity search, the 4 sequences from Rajshahi showed the highest nucleotide similarity (96.67%, 96.68%, 96.67%, 96.66%) with a CDV sequence (MK037461) previously identified from Asiatic lions (*Panthera leo persica*) from India in 2018^20^. The single sequence from Chattogram showed the highest nucleotide similarity (99.06%) with a CDV sequence (MW876862) previously identified from an Indian jackal (*Canis aureus indicus*) in 2019.

### Phylogenetic analysis

**Figure 1:**
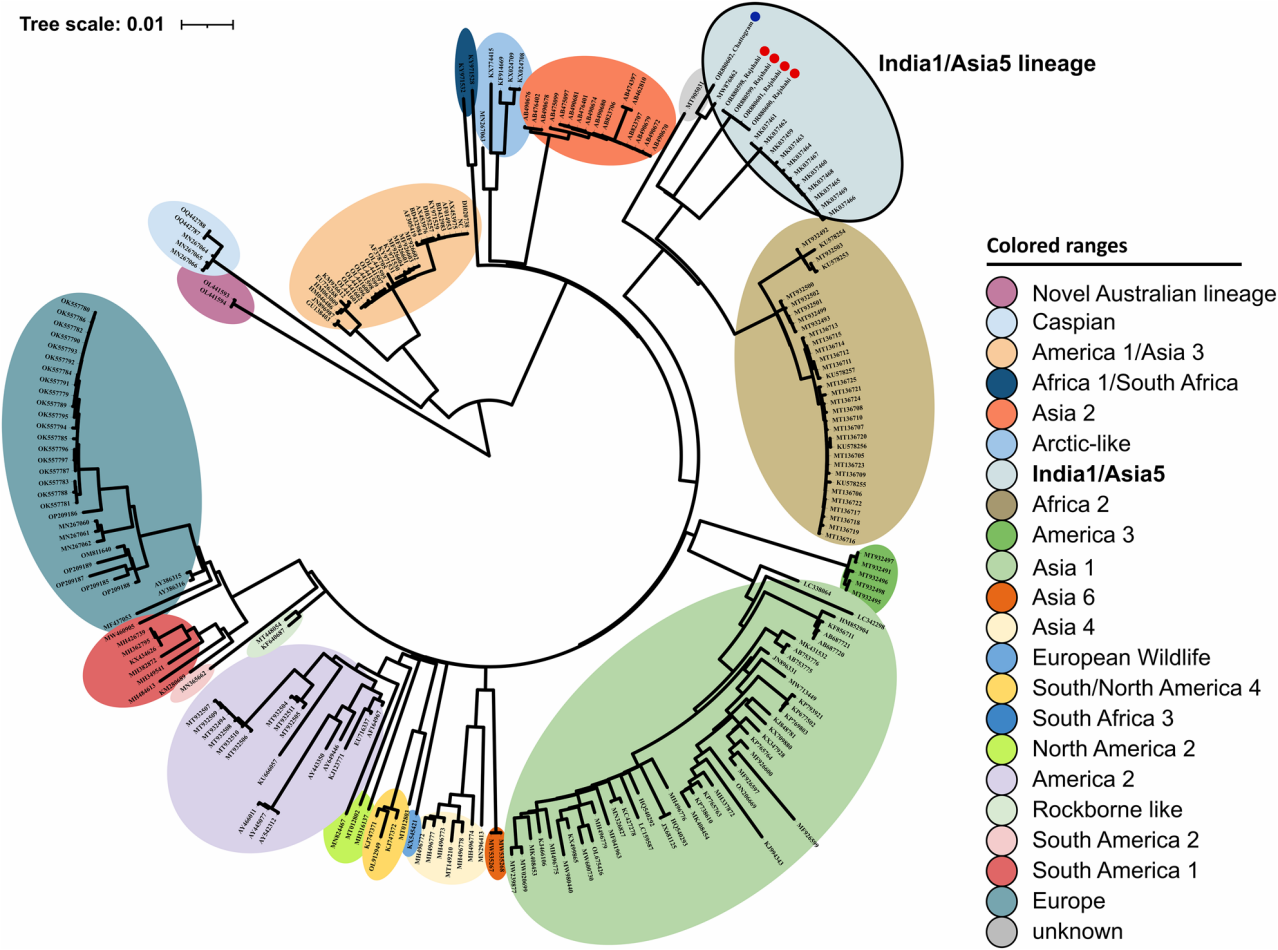
Maximum likelihood phylogenetic tree based on 240 CDV complete or near-complete genomes. Phocine distemper virus (PDV) (GenBank accession number: KY629928) was used as an outgroup to root the phylogenetic tree. The novel sequences from Bangladesh are indicated with dots (red: from Rajshahi, blue: from Chattogram).

Based on the phylogenetic analysis comparing a large dataset of complete or near-complete CDV genomes, the novel sequences from Bangladesh clustered into the India-1/Asia-5 lineage (Fig. 1). Summing up the regional sequence data, we report the presence of this CDV lineage in two distant cities within Bangladesh. These novel sequences from stray dogs are positioned on separate branches within the India-1/Asia-5 lineage cluster, according to their city of origin (Fig. 1, highlighted by blue and red dots). Interestingly, they are positioned within this lineage, close to previously reported CDV sequences from India Asiatic lions and Indian jackal, supporting the hypothesis of interconnected interface of animal diseases between human-related stray dogs and wildlife.

## Discussion

We confirmed the presence of CDV in Bangladesh using real-time RT-PCR and Oxford Nanopore sequencing in a mobile, on-site laboratory setup. To our knowledge, these are the first CDV sequences from Bangladesh. During sample collection and processing, we worked together with local experts to transfer knowledge about diagnostic solutions for CDV detection. Further data is essential to obtain a more in-depth understanding of the role of stray dog-driven CDV evolution and its real impact on spillover host populations.

Based on available literature data, CDV might be a common disease among stray dogs in Bangladesh. However, the genetic characteristics of these circulating strains are to date unknown. In Bangladesh based on clinical signs and diagnosis, CDV was reported in dogs from different part of the country^21,22,24–27^. The underlying factors in this urban ecosystem, which are driving the spatial–temporal presence, impact and genetic factors of the virus are unknown. This constitutes a significant risk factor for the growing culture of keeping household pets in the country. Apart from domesticated animals, in 2010, 2 out of 5 apparently healthy golden jackal (*Canis aureus*) were positive using CDV specific RT-PCR, from Mymensingh. In this regard, the authors raised its importance, specific RT-PCR for the detection of CDV can significantly contribute to the diagnosis of the virus. Furthermore, to learn about the epidemiology of CDV, it is necessary to sequence the genome of the virus and test a large number of samples^23^.

Here we report the presence of the India-1/Asia-5 lineage in distant regions of Bangladesh. This lineage was identified from South Asian region during the recent years. Related sequences are available from dog, palm civet cat, Indian jackal, and leopard from India from 2015–2016 and 2019, and a leopard from Nepal from 2016 and 2019^19^. In 2018, CDV was detected in wild and captive carnivores; n = 68 Asiatic Lions and n = 6 leopards, and it was confirmed by complete genome sequencing that CDV belongs to the India-1/Asia-5 lineage^20^. Based on available data from the region, the India-1/Asia-5 lineage is affecting both the human-associated stray dog population and through frequent contact events multiple vulnerable or protected animal species. Therefore, the general understanding of the most abundant stray dog population as a key player in the maintenance of CDV may greatly facilitate conservation efforts of other susceptible animals in the region. Genomic surveillance with PCR and sequencing is the most powerful tool to understand viral spreading and evolution patterns. There are multiple sequence technologies, among which the Oxford Nanopore Technology is a fast and efficient procedure, with the advantage of high mobilization capacity^7^.

Vaccination against CDV is an effective prevention strategy, especially in regions with low vaccination rates. The study highlighted the importance of regulating the population of stray dogs, implementing spaying and neutering programmes, and vaccinating them against the virus. More studies are needed to reveal the presence and genetic attributes of CDV in Bangladesh and understand the risk of cross-species transmission events between street dogs and other carnivores, such as the Bengal tiger or golden jackal. While CDV is primarily known as an animal pathogen, recent studies have raised concerns about its potential to infect humans, particularly in communities with low measles vaccination rates and frequent exposure to CDV-positive animals^37–39^. In vivo experimentally infected cynomolgus macaques, Measles virus vaccination induces partial protection against CDV challenge infection^39^. It can be a concern that, CDV can infect nonhuman primates rhesus macaques (*Macaca mulatta*) and cynomolgus macaques (*Macaca fascicularis*) naturally^40,41^, these animals can also be found in naturally in Bangladesh. This emphasizes the importance of conducting snapshot studies in low-resource settings to quickly gain insights into the genomic characteristics of circulating variants.

The limitations of the study include the difficulty in individual-level tracking of stray dogs, as there was no opportunity for follow-up sampling. During the sampling process, there was limited opportunity for a thorough clinical examination of the dogs. Additionally, due to the low viral genomic copy number in some of the samples, we were not able to obtain complete genome sequences. Further studies are essential to better understand the transmission patterns of CDV within stray dog populations and to accidental spillover hosts in Bangladesh.

In summary, this study demonstrated the importance and utility of mobile genomic surveillance studies for rapid insight into the epidemiological situation of animal diseases in low resource regions, potentially useful both for domestic animals and wildlife. To maintain a long-term solution for the complexity of CDV problem (risk for wildlife, conservation of protected species, risk for pet culture) a comprehensive surveillance system would be beneficial. For laying the foundations of such programme, mobile surveillance solutions are optimal, regarding their cost-efficiency and rapidity for generating baseline data. The study was conducted in collaboration with local experts, aiming to transfer knowledge and improve the diagnosis of CDV in stray dogs in Bangladesh. The reliability of mobile animal disease diagnostics is beyond CDV and are potentially applicable for other prevalent diseases in the future, such as peste des petit ruminants or rabies.

## Methods

### Sample collection

**Figure 2:**
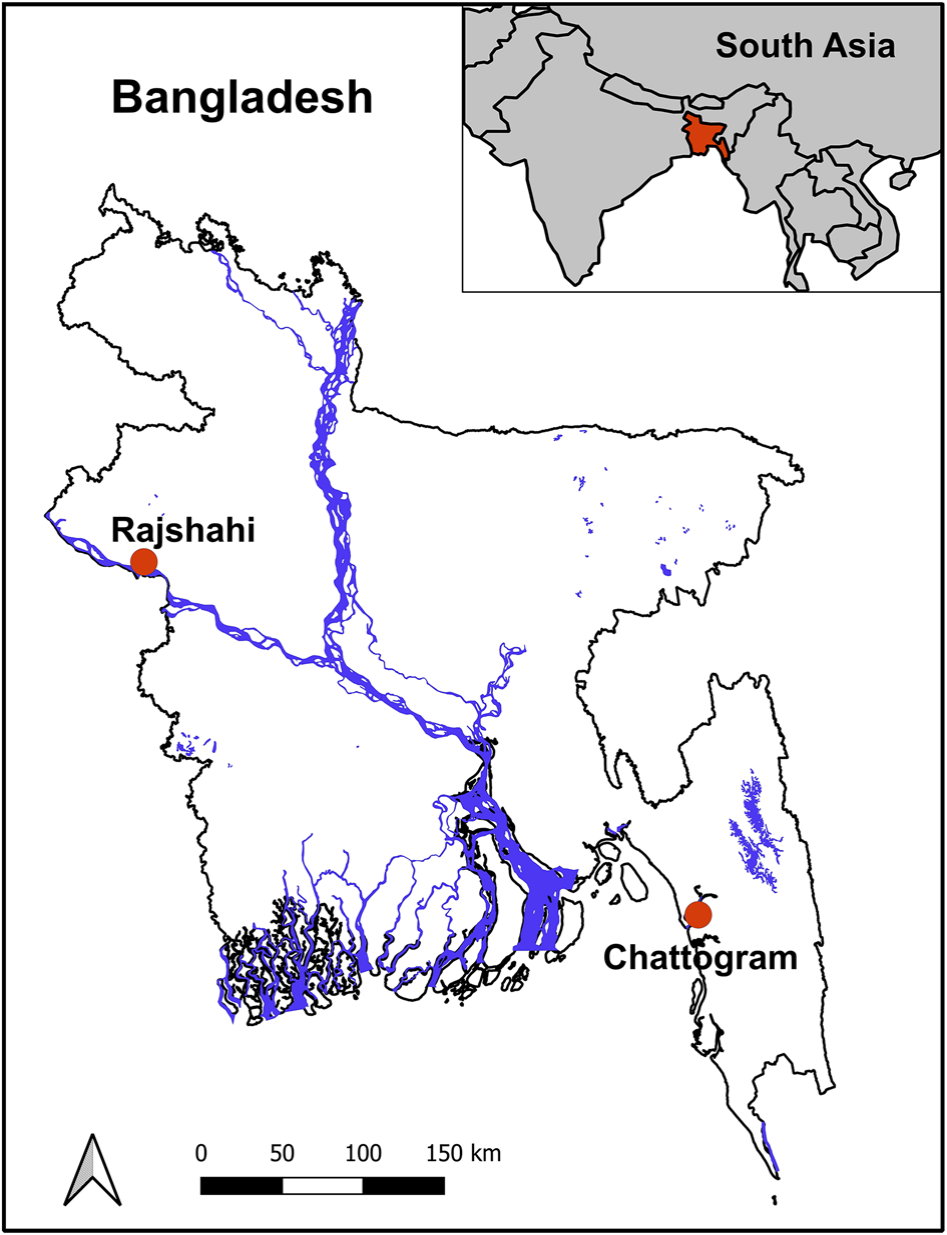
Map of Bangladesh with the indication of sampling sites with red dots.

We collected 355 saliva samples from stray dogs between 20 and 28 February in Rajshahi (n = 135) and between 4–5 March and 16–20 June in Chattogram (n = 220) (Fig. 2). These stray dogs are regularly fed by local communities; therefore, they can be sampled without trapping or catching with nets. The dogs were petted, fed, and sampled during feeding. The oral swabs were resuspended into 500 μl of phosphate-buffered saline (PBS) solution. In the case of morning collection, the samples were processed immediately on the day of collection, whilst in the case of evening collection, they were stored the next day at − 20 °C until processing.

The sampling procedures were conducted under the oversight of the veterinary clinics at University of Rajshahi and Chattogram Veterinary and Animal Sciences University. These clinics are involved in several initiatives for stray dogs, including vaccination and neutering programs. Our sampling, which involved non-invasive saliva collection, was carried out with the assistance of professional veterinary practitioners of these clinics.

### Nucleic acid extraction and PCR reactions

The total RNA was extracted using Direct-Zol RNA MiniPrep (Zymo Research, USA). The samples were screened with a CDV-specific real-time RT-PCR using the QIAGEN OneStep RT-PCR kit (Qiagen, Germany) using CDV-F 5ʹ-AGCTAGTTTCATCTTAACTATCAAATT-3ʹ and CDV-R 5ʹ-TTAACTCTCCAGAAAACTCATGC-3ʹ primers, and CDV-Pb 5ʹ-FAM-ACCCAAGAGCCGGATACATAGTTTCAATGC-TAMRA-3ʹ probe^42^ with the reaction setup as follows: at 50 °C for 30 min, and 95 °C for 15 min, followed by 45 cycles of 95 °C for 20 s, 46 °C for 30 s 72 °C for 30 s (the fluorescence signal was detected during the annealing step). All real-time RT-PCR were run on the MyGo Mini PCR system platform (IT-IS Life Science, Ireland) and analysed with the MyGo PCR software (v.3.5.2). RT-PCRs were performed immediately after RNA extraction without freeze-thawing the nucleic acid, avoiding RNA degradation.

### Nanopore sequencing

We amplified the complete coding region of the genome with CDV-specific amplicon-based sequencing method optimized for MinION (Oxford Nanopore Technologies, UK)^7,35,36^. SuperScript IV (Invitrogen, USA) was used to generate single strand cDNA using random hexamers. Amplicons were generated from cDNA with Q5 Hot Start HF Polymerase (New England Biolabs, USA), with two different primer sets in parallel pools to obtain the best possible genome coverage with different amplicon setup (CDV_1000bp pool 1 and 2, CDV_2000bp pool 1 and 2). PCR products were purified using the AMPure XP beads (Beckman Coulter, USA). PCR amplicons pools were end-repaired and dA-tailed with the NEBNext Ultra II End Repair/dA-Tailing Module (New England Biolabs, USA). 1.5 µL end-prepped DNA was transferred to the next reaction and the barcodes derived from EXP-NBD196 (Oxford Nanopore Technologies, UK) were ligated with NEBNext Ultra II Ligation Module (New England Biolabs, USA). All the pooled barcoded libraries were purified using Ampure XP beads (Beckman Coulter, USA), the AMII sequencing adapters were ligated with NEBNext Quick Ligation Module. The quantity of the final library was measured with Qubit dsDNA HS Assay Kit (Invitrogen, USA) on a Qubit 3 fluorometer. 75 ng final libraries were loaded onto a R9.4.1 (FLO-MIN106D) flow cell. The detailed protocol can be found on the protocols.io website^43^.

### Data analysis

The raw sequencing data were basecalled using guppy (ONT guppy v6.4.6.) high accuracy basecaller algorithm (dna_r9.4.1_450bps_hac config file). From the fastq files, only reads with a minimum Q score of 10 were selected for the analysis. Demultiplexing and trimming of barcodes were performed also with guppy using default parameters of ‘guppy_barcoder’ runcode. Primers were trimmed and the reads were filtered on the expected length of amplicons with Geneious Prime (v2023.0.4) software. To generate a consensus sequence, the processed reads from all samples were mapped to the CDV genome (NCBI accession number: MT932501) with the usage of MiniMap 2.24. The generated consensus sequences were manually checked for basecalling errors.

### Phylogenetic analysis

In the phylogenetic analysis, 5 near-complete genomes from the 12 positive samples were included. Prior to the phylogenetic reconstruction, cognate sequences were retrieved from the GenBank database and aligned with our these sequences using the MAFFT alignment web-server^44^. The final dataset comprised 240 near-complete genomes, referring to all cited CDV lineages. IQTree webserver was used for both best substitution model selection and maximum likelihood phylogenetic tree reconstruction using 1000 bootstrapping and GTR + F + I + R3 model^45^. The final tree was edited with the iTOL online tool (iTOL, Heidelberg, Germany)^46^. In addition, the obtained nucleotide sequences were feed to the NCBI BLASTn web tool to evaluate identities with cognate sequences.

### Ethics declarations

The samples were collected in a non-invasive manner. The work was carried out under the framework of the permission from the Ethical Committee of Chattogram Veterinary and Animal Sciences University. Approval No: CVASU/Dir(R&E)EC/2022/405/12.

## Data Availability

All the sequences generated have been deposited in NCBI GenBank database with accession numbers OR880598-OR880602. The datasets generated during and/or analysed during the current study are available from the corresponding author upon request.
